# What Are the Contributions of Handedness, Sighting Dominance, Hand Used to Bisect, and Visuospatial Line Processing to the Behavioral Line Bisection Bias?

**DOI:** 10.3389/fpsyg.2018.01688

**Published:** 2018-09-12

**Authors:** Audrey Ochando, Laure Zago

**Affiliations:** ^1^UMR 5293, Institut des Maladies Neurodégénératives, University of Bordeaux, Bordeaux, France; ^2^UMR 5293, Centre National de la Recherche Scientifique, Institut des Maladies Neurodégénératives, Bordeaux, France; ^3^UMR 5293, CEA, Institut des Maladies Neurodégénératives, Bordeaux, France; ^4^UMR 5293, Team 5: GIN Groupe d’Imagerie Neurofonctionnelle, Centre Broca Nouvelle-Aquitaine, Institut des Maladies Neurodégénératives, Bordeaux, France

**Keywords:** spatial attention, deviation bias, healthy participants, handedness, hand dominance, eye sighting dominance

## Abstract

In a sample of 60 French participants, we examined whether the variability in the behavioral deviation measured during the classical “paper and pencil” line bisection task was explained by individual laterality factors such as handedness and eye sighting dominance, as well as the hand used to bisect, and the spatial position of the line to bisect. The results showed the expected main effects of line position and hand used to bisect, as well as some interactions between factors. Specifically, the effect of the hand used to bisect on the deviation bias was different as a function of handedness and line position. In right-handers, there was a strong difference between the biases elicited by each hand, producing a hand-used asymmetry, observed for each spatial position of the line. In left-handers, there was no difference in deviation as a function of hand used to perform the bisection, except when all factors triggered attention toward the left side such as bisecting left-displaced lines, with the left dominant hand, producing a strong leftward deviation as compared to the reduced bias exhibited with the right non-dominant hand. Finally, the eye sighting dominance interacted with handedness and line position. Left-handers with a right sighting dominance showed a leftward bias when they bisected left-displaced lines, while right-handers with a left sighting dominance showed an inversed bias when they bisected rightward lines. Taken together, these findings suggest that the behavioral deviation bias relies on the integration of the hemispheric weights of the visuospatial processing of the stimuli, and the motoric component of the hand used to bisect, as well as those linked to individual laterality factors. When all these factors producing asymmetric cerebral activation coincide in the same direction, then their joint effect will provide the strongest asymmetric behavioral biases.

## Introduction

In neurologically intact individuals, much research has shown the existence of perceptual asymmetries during free-viewing conditions (for a review, Voyer et al., 2012). For example, during the “paper and pencil" line bisection task, usually used to assess hemispatial neglect (Heilman et al., 1985; Doricchi and Angelelli, 1999; Sperber and Karnath, 2016), non-clinical population exhibits a small but consistent tendency to slightly mark to the left of the veridical midpoint (Jewell and McCourt, 2000), referred to as pseudoneglect (Bowers and Heilman, 1980). This attentional orientation toward the left hemispace would be related to the asymmetrical control of spatial attention over the hemispheres (Mesulam, 1981; Kinsbourne, 1987; Driver and Vuilleumier, 2001; Corbetta and Shulman, 2011), with a stronger activation of the right hemisphere, being the dominant hemisphere for visuospatial attentional function (Kinsbourne, 1970; Mesulam, 1999), leading to an over-representation of the left side of space and a shift of the subjective center of the line toward that side. Recently, we demonstrated using fMRI during a line bisection judgment (LBJ) task that the degree of leftward behavioral bias was associated with the degree of rightward hemispheric lateralization (Zago et al., 2017).

Although this behavioral leftward bias is a reliable phenomenon (Jewell and McCourt, 2000), inter-individual variability exists both in the magnitude and the direction of the bias (Manning et al., 1990), and the demonstration of the factors that contribute to the variability of this bias remains open. For example, some studies have shown that visuospatial factors, such as the direction of the visual scanning of the line (Chokron and Imbert, 1993; Brodie and Pettigrew, 1996), the visual hemispace, and the hemispatial body field in which the line is presented (Bowers and Heilman, 1980; Luh, 1995; Mennemeier et al., 1997; McCourt, 1999) contribute to the behavioral attentional bias. When the visual scanning is initially performed from the left, it induced greater leftward deviations compared to right scanning (Brodie and Pettigrew, 1996; Chokron et al., 1998). In addition, lines positioned in the left hemispace induced more asymmetrical bisection than right-displaced lines (Luh, 1995; Mennemeier et al., 1997; McCourt, 1999). These visuospatial factors (visual scanning to the left and visual stimulation of left hemispace) would additionally activate the right hemisphere, and probably induce a stronger behavioral asymmetry.

In addition to these visuospatial components, the line bisection task also involves manual/motor components that are lateralized. Specifically, the hand used to perform the bisection and the handedness of the participants are two interacting (and sometimes confounding) factors that have been shown to modulate the bias (for a review, see Jewell and McCourt, 2000; McCourt et al., 2001; Hausmann et al., 2003). For example, the study of Luh (1995) reported a stronger leftward bias in left-handers than in right-handers (Luh, 1995), but both groups used their dominant (or preferred) hand to give the response, confounding the effects of handedness and hand used. The study of Scarisbrick et al. (1987) reported that left-handers using their left hand showed greater leftward bias during a visual line bisection task than right-handers using their left non-dominant hand (Scarisbrick et al., 1987). A greater leftward deviation bias was found in right-handers using their left non-dominant hand compared to their right preferred hand (Brodie and Pettigrew, 1996), while left-handers bisected horizontal lines toward the left when using the left dominant hand and more toward the right when using their right hand (Bradshaw et al., 1987; Dellatolas et al., 1996). Because each hand is controlled primarily by the contralateral hemisphere, the hand effect may reflect activation of the right-hemispheric sensorimotor areas for control of the left-hand, leading to higher global activation for the right hemisphere than the left hemisphere, which in turn leads to a larger leftward bias during bisection for left-hand use compared to right-hand use. In addition, as suggested by Brodie and Dunn (2005), moving the non-dominant hand (or non-preferred hand) during line bisection would require more conscious effort associated with extended and/or bilateral cortical activation that may modify the deviation bias (Brodie and Dunn, 2005).

Concerning the cortical organization of motor control, some studies tended to show differences between left- and right-handers. For example, Solodkin et al. (2001) reported that left-handers showed less brain lateralization than right-handers during a sequential movement task (Solodkin et al., 2001, but see Kroliczak et al., 2016). More recently, Tzourio-Mazoyer et al. (2015) demonstrated a different pattern of deactivation between left- and right-handers within the ipsilateral motor cortex during movement of the dominant hand (Tzourio-Mazoyer et al., 2015). Lastly, a rightward bias was found in left-handers using their preferred hand and when adopting a visual scanning from right to left (Brodie and Dunn, 2005), indicating an interaction between these factors. Taken together, these results suggest that the difference between left-handers and right-handers should be carefully investigated during line bisection task, and that the variability of the deviation bias would probably be explained by the interaction between manual/motoric and visuospatial factors.

Finally, eye sighting dominance, defined as the behavioral preference for one eye over the other under monocular viewing conditions (Coren and Kaplan, 1973; Porac and Coren, 1976; Coren, 1993) is an underscored individual laterality factor that could also contribute to the deviation bias during this visuomotor task. Although, it is known that each cerebral hemisphere processes information coming from the contralateral visual hemifield, recent imaging studies indicated that the ocular dominance is regulated by the ipsilateral occipital cortex. Specifically, the visual cortex ipsilateral to the dominant eye has been shown to be larger in size than the contralateral cortex (Erdogan et al., 2002), and the magnitude of the V1 response in the ipsilateral visual cortex of the dominant eye is greater during the stimulation of the dominant eye than that during the non-dominant one (Shima et al., 2010). Beyond the visual cortex, we observed using fMRI during a visually-guided saccade task that the rightward cerebral asymmetry of the dorsal frontoparietal attention network was more pronounced in left-handed participants with a right eye sighting dominance (i.e., eye/hand crossed) during basic shifts of attention in a visually guided saccade task (Petit et al., 2015). If the eye dominance induced stronger activation of the ipsilateral occipital cortex, this increased rightward cerebral lateralization could be the consequence of the functional connection between visual input and motor output within the right hemisphere for left-handers with a right sighting-eye (Petit et al., 2015). Finally, Chaumillon et al. (2014) demonstrated that this particular relationship between sighting dominance and ipsilateral cortex resulted in a shorter manual reaction times in response to lateralized visual target appearing in the contralateral visual hemifield with respect to the dominant eye (Chaumillon et al., 2014). Taken together, these findings highlight the need to consider eye dominance in studies investigating the processes underlying visuomotor actions, such as during line bisection.

The aim of the present study was to evaluate the effects of individual laterality factors such as handedness and eye sighting dominance, as well as of visuospatial and motoric factors related to the task to be performed, on the line bisection deviation bias. Specifically, in a sample of French participants enriched in left-handers, we examined whether the variability in the behavioral deviation measured during the classical “paper and pencil” line bisection task was explained by handedness, the hand used to bisect, eye sighting dominance, and the spatial position of the line to bisect. Based on the hypothesis that the amount of behavioral deviation bias obtained during line bisection would reflect the amount of cerebral activity elicited by the integration of different spatial attentional, motoric and visuospatial processes involved in the task, we predicted that the combination of these different factors would elicit stronger leftward deviation, such as in left-handers, who bisect left-displaced lines with their left dominant hand, perhaps with a right-eye sighting dominance. *A* *contrario*, we would expect to find a reduced leftward bias and even a rightward bias in right-handers who bisect right-displaced lines with their right dominant hand.

## Materials and Methods

### Participants

We recruited 92 healthy volunteers, measured their hand preference and eye-sighting dominance (ESD), as well as the line deviation bias during a “paper and pencil” line bisection task. All participants provided written informed consent to participate in the experiment, and the protocol was approved by the ethics committee of Bordeaux University.

In the present study, due to the acknowledged difference in manual preference strength between right- and left-handers, we selected 60 participants with a strong (right or left) hand preference (see below). The mean age of this study sample was 22.9 years (*SD* = 3.5; range: 18–34 years).

#### Hand Preference

Hand preference was assessed using the Edinburgh inventory questionnaire (EI, Oldfield, 1971). Based on the distribution of EI scores obtained in a sample of 92 participants, we retained the upper (EI score ≥ to +85) and lower (EI score ≤ to −65) third of the population. Thus, the 29 selected left-handers (LH, 14 women) had a mean EI score of −81.2 (*SE* = 17.4), and the 31 right-handers (RH, 16 women) had a mean EI score of 97.8 (*SE* = 4.6). Note that there was a significant difference in the absolute EI value between the two groups [*t*(58) = 5.1, *p* < 0.0001] due to both lower values and greater variability in the LH than in the RH. In addition, note that the sample of participants of this study is not representative of the general population, as it was deliberately enriched in left-handers.

#### Eye-Sighting Dominance

Eye-sighting dominance (ESD) was evaluated for each participant using a variation of the hole-in-the-card test (Durand and Gould, 1910). The participant was asked to extend his/her arms in front of him/her and to form a diamond-like frame using the thumb and index finger of both hands, replacing the card’s hole to see through. He/she was then requested to stare through this frame at a specific object located at distance. Without moving his/her hands, the participant then had to look at the object using only one eye, first the right and then the left. The preferred sighting eye was determined to be that for which vision was the same as when looking with both eyes open. Note that using both hands to form a diamond-like frame avoids any bias due to a sighting measure using a single hand.

Among the 29 LH, 20 participants (including 9 women; mean age = 21.8 ± 3.2) showed a left eye-sighting dominance (L-ESD) and 9 (including 5 women; mean age = 23.4 ± 4.3) showed a right ESD (R-ESD). Among the 31 RH, 21 participants (including 11 women; mean age = 23.0 ± 3.2) had a R-ESD, and 10 (5 women; mean age = 24.7 ± 3.4) had a L-ESD.

### Line Bisection Task: Procedure and Materials

The line bisection task contained 17 horizontal black lines of 1 mm width distributed on a white sheet of paper (landscape presentation), with a distance of 18 mm away from the left/right and upper/lower margins of the page. The lines that we used were similar to those used in the study of Hausmann et al. (2002). The lines ranged from 80 to 240 mm in length, in steps of 20 mm. They were pseudo-randomly positioned so that seven lines appeared in the middle of the sheet (one line of 100, 160, and 180 mm, two lines of 200 mm, and two of lines of 220 mm), and 10 lines were positioned either to the rightmost (five lines) or leftmost regions of the sheet (one line each of 80, 120, 140, 180 , and 240 mm). The sheet was laid in front of the participant’s midline. Participants were instructed to bisect all lines into two parts of equal length by marking the subjective midpoint of each line with a fine pencil. All participants bisected the lines from the top to the bottom of the page, and each line was covered after bisection. Participants completed the task twice, with their right and left hands. The order was counter-balanced across participants. There was no time restriction. The deviations to the left or to the right of the center were carefully measured to 0.5 mm accuracy. The percent deviation bias was computed using the following formula of Hausmann et al. (2002): [(measured left half – true half)/true half] × 100. The mean deviation bias was computed for each position of the lines (left, middle and right), separately for each hand used. Negative values indicate a left bias, whereas positive values indicate a bias toward the right.

### Statistical Analyses

Statistical analyses were conducted using the ezANOVA function of the ez R-package (version 4.4-0)^1^. The deviation bias was analyzed with a mixed-model analysis of variance with repeated measures including “Eye sighting dominance” (ESD) and “Handedness” as between-subjects factors and “Hand used” (Left, Right) and “Line position” (left, middle, and right) as within-subjects factors. All *post hoc* comparisons were corrected for multiple comparisons using Bonferroni correction. Standardized effect size expressed in terms of generalized eta-squared (eta_G_^2^) value are reported (Bakeman, 2005). Effect sizes were characterized as small (0.01 < eta_G_^2^ < 0.06), medium (0.06 < eta_G_^2^< 0.14) or large (0.14 < eta_G_^2^) according to published recommendations (Lakens, 2013).

## Results

### Analysis of Deviation Bias

The distribution of mean deviation bias followed the Gaussian law (Shapiro-Wilk, *W* = 0.9, *p* = 0.2). On average, the deviation bias was −0.5 ( ± 2.2), indicating pseudoneglect [one-tailed *t*-test against zero, i.e., no bias; *t*(59) = −1.7, *p* = 0.04] over the sample of 60 participants. In terms of distance, the bisection mark was placed approximately 0.50 mm to the left of the true center (range in mm; −12.1; +11.4).

**FIGURE 1:**
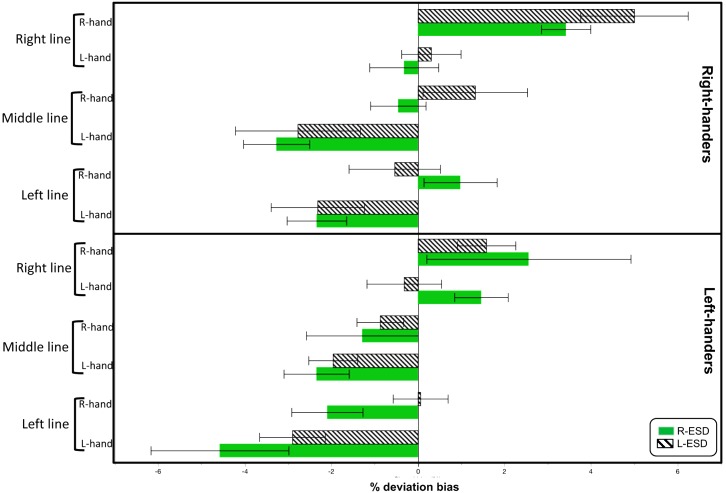
Mean deviation bias (in %) during line bisection as function of Handedness, Eye sighting dominance (ESD), Hand used to bisect (L-hand and R-hand), and Line position (left, right, and middle).

Descriptive statistics for each condition are shown in **Figure 1**. To test for the existence of pseudoneglect, we calculated Bonferroni-adjusted one-sample *t*-tests for each of the 24 different conditions (threshold of *p* < 0.002 Bonferroni-adjusted). Significant pseudoneglect was found in LH with L-ESD and in RH with a R-ESD when both groups bisected left-displaced and middle positioned lines with their left hand. Significant rightward deviation was found in RH with a R-ESD when they bisected right-displaced lines with the right hand.

Note that the largest values of leftward and right biases were found in LH with a R-ESD bisecting left-displaced lines with the left hand, and in RH with a L-ESD bisecting right-displaced lines with the right hand, respectively. However, these values did not reach the Bonferroni-adjusted threshold (LH R-ESD Left-hand left-line: *p* = 0.02, *n* = 9; RH L-ESD Right-hand right-line: *p* = 0.003, *n* = 10).

The ANOVA revealed two main effects. First, a “Line position” effect [*F_(_*_2,112)_ = 36.3, *p* < 0.0001; eta_G_^2^ = 0.15] showed that leftmost displaced lines (mean ± SD; −1.4 ± 2.9) and middle lines (−1.5 ± 2.5), both produced leftward deviation bias different from the rightward bias elicited by the rightmost displaced lines (+1.5 ± 3.1). Second, a “Hand used” effect [*F*_(1,56)_ = 45.0, *p* < 0.0001; eta_G_^2^ = 0.13] indicated that the left-hand elicited a leftward bias (−1.8 ± 2.6), while the right hand elicited an inversed bias (+0.8 ± 2.8).

**FIGURE 2:**
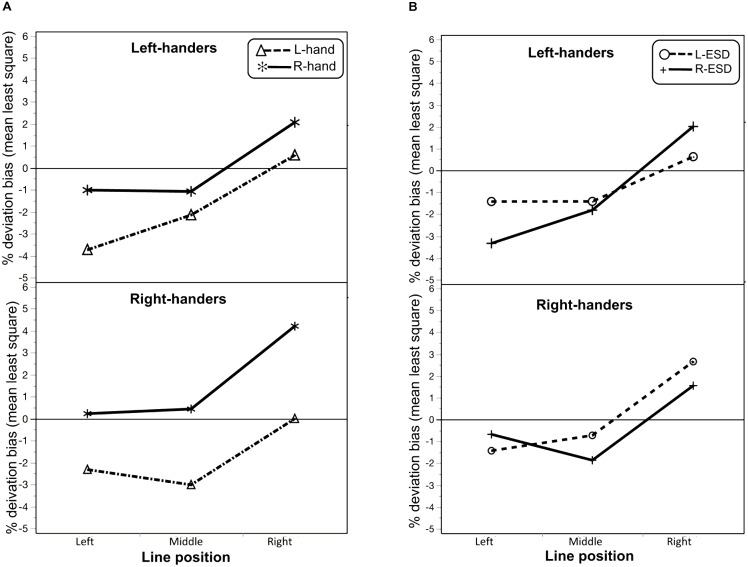
**(A)** Interaction plot between the line position and the hand used to bisect (L-hand and R-hand) in left-handers and right-handers on deviation bias. **(B)** Interaction plot between the line position and Eye sighting dominance (L-ESD and R-ESD) in left-handers and right-handers on deviation bias.

In addition, the ANOVA revealed three interactions. One interaction was found between “Handedness” × “Hand used” [*F*_(1,56)_ = 3.8; *p* = 0.05; eta_G_^2^ = 0.01] and the other was found between “Handedness” × “Hand used” × “Line position” [*F*_(2,112)_ = 5.4; *p* = 0.005; eta_G_^2^ = 0.008]. As illustrated in **Figure 2A**, this last interaction indicated that a differential effect of the hand used to bisect between LH and RH on deviation bias as a function of line position. To further investigate this interaction, we computed *post hoc* comparisons Bonferonni-adjusted, revealing that, in LH, the difference between the left and right hand on deviation bias was significant for left-displaced lines only (*p* = 0.01), while for RH the difference between hands was significant for each line position (all *p*’s < 0.0001).

A third interaction was found between “Handedness” × “ESD” × “Line position” [*F*_(2,112)_ = 4.5; *p* = 0.01; eta_G_^2^ = 0.02; **Figure 2B**], indicating a different effect of ESD between LH and RH as a function of line position. *Post hoc* comparisons Bonferonni-adjusted indicated that this difference between L-ESD and R-ESD was significant in RH when participants bisected middle lines (*p* = 0.04), with larger pseudoneglect for RH with a R-ESD. This difference was close to significance for right-displaced lines (*p* = 0.06), with RH with a L-ESD tended to show a larger rightward deviation that the one observed in RH with a R-ESD.

## Discussion

The aim of the present study was to evaluate the respective contributions of the visuospatial processing of the stimulus (here, the spatial position of the lines), the hand response (the hand used to bisect) as well as individual laterality factors such as the sighting dominance and the handedness to explain the variability of the behavioral attentional bias measured during the paper and pencil line bisection task.

First, the results demonstrated that, over the sample of participants, this free-viewing line bisection task produced a leftward deviation bias, congruent with the pseudoneglect well documented in the general population, as a result of right cerebral hemisphere dominance for spatial attention (Zago et al., 2017). As concerns the variability of this bias, the results of this study indicated a strong impact of both the spatial position of the stimulus and the hand used to bisect on the direction and magnitude of the deviation bias. In addition, these results also indicated that the variability of the deviation bias was also explained by interactions between individual laterality factors such as handedness and sighting dominance, and visuospatial and hand motoric variables related to the performance of the task.

These results replicate those of previous studies showing that the deviation bias is strongly affected by the position of the lines on the sheet as well as the hand used to perform the bisection (for a review, see Jewell and McCourt, 2000). More specifically, the results showed that both centered lines and left-displaced lines produced pseudoneglect, suggesting that these two positions triggered attention toward the left-side of space. By contrast, bisecting right-displaced lines reduced pseudoneglect, and produced an inversed bias. These findings are consistent with the behavioral results of a recent study measuring line bisection deviation in a large sample of more than 500 participants (Ocklenburg et al., 2018). Similarly, using the left hand induced greater leftward deviations than the right hand. Together, these findings demonstrated that the direction of the deviation bias is strongly triggered by the side of the visuospatial stimulation, as well as by the side of the motoric hand response, reinforcing the hypothesis of the visuospatial and motoric origins of pseudoneglect.

The interesting contribution of the present study is that the deviation bias was also modulated, although to a lesser extent, by individual laterality factors, indicating, as suggested by Learmonth et al. (2015), that the deviation bias is “a multicomponent phenomenon.” As concerns handedness, the results demonstrated a different effect of hand used in function of line position between LH and RH. Specifically, in RH, each hand produced different deviation biases for each of the spatial position of the lines. By contrast, in LH, there was no difference in deviation bias between hands, except for left-displaced lines, for which the left dominant hand produced a strong leftward deviation that was significantly larger than the reduced bias exhibited with the non-dominant hand. Except for this specific condition, LH exhibited a lack of asymmetry between the hands that could be linked to the well-known lower behavioral hand-lateralization phenomenon observed in left-handers. Indeed, left-handers appear to have a lower difference in manual ability between their dominant and non-dominant hand due to a relatively preserved ability of their non-dominant hand, compared to the non-dominant hand of right-handers (Tzourio-Mazoyer et al., 2015). Even if we selected participants with a strong hand preference on the EI score, the group of left-handers still displayed a lower manual preference strength and larger variability than the group of right-handers. This lower manual asymmetry in LH was also expressed during this visuospatial/motor attentional task, during which the hand used factor has a lower impact than in RH. However, when all factors drive attention to the left (left dominant hand and left-displaced line), then the degree of pseudoneglect was maximized, suggesting a joint effect of these factors.

As concerns eye sighting dominance, the results indicated that this factor showed a subtle effect, evidenced in specific conditions, such as when individuals have a crossed hand-eye dominance. Specifically, the highest mean value of pseudoneglect was observed in LH with a right sighting dominance when they bisected left-displaced lines with the left-hand. In return, the highest value of inversed pseudoneglect was found in RH with a left sighting dominance when they bisected rightward lines with their dominant hand. Although additional studies are needed to confirm these observations by including a larger number of individuals with a crossed hand/eye dominance (Bourassa et al., 1996), these findings would suggest an additive combination of the hemispheric weights related to the different factors. If, as suggested by previous studies (Erdogan et al., 2002; Shima et al., 2010), eye dominance is predominantly controlled through the ipsilateral occipital cortex, then all factors of these two conditions would put activation weights on the same cerebral hemisphere, triggering attention contralateral to the most activated hemisphere.

Further neuroimaging investigations are now needed to understand the underlying cerebral mechanisms of this behavioral deviation bias. Based on our previous neuroimaging studies showing an association between the strength of the pseudoneglect and rightward cerebral asymmetries (Zago et al., 2016, 2017), and the evidence of right occipito-temporal regions underlying pseudoneglect measured with a perceptual line bisection judgment (Zago et al., 2017), we suggest that the specific condition that elicit the strongest behavioral pseudoneglect would also be associated with strongest rightward hemispheric asymmetries. It remains to further explore the respective cortical contributions related to hand and sighting dominance to explain inversed pseudoneglect.

## Conclusion

The present work demonstrated that the variability of the behavioral bias measured during the line bisection task is explained by the integration of different factors related to the visuospatial processing of the stimuli and the motoric component of the hand used to bisect, as well as some individual laterality factors. When all these factors producing asymmetric cerebral activation coincide in the same direction, then their joint effect will provide the strongest asymmetric behavioral biases.

## Author Contributions

LZ designed the study, analyzed the data, and wrote the paper. AO performed the experiments.

## Conflict of Interest Statement

The authors declare that the research was conducted in the absence of any commercial or financial relationships that could be construed as a potential conflict of interest.

## Footnotes

1 https://cran.r-project.org/web/packages/ez/index.html
